# Supplementary material for: Content-rich biological network constructed by mining PubMed abstracts
Source: BMC Bioinformatics. 2004 Oct 8;5:147. doi: 10.1186/1471-2105-5-147 (PMC528731; doi:10.1186/1471-2105-5-147)
Supplement: Additional File 2 — The original results of the above study (non-essential files are deleted to keep the file size under the limit set by BMC bioinformatics). [file 1471-2105-5-147-S2.bz2 › chilibotAdditionalFile2/dip50/347ID7721786E4495/html/IL4_IL4R.html]

 


 **IL4** and **IL4R** 
  
Found 5329 abstracts in PubMed, retrieved 50.  
 

 What does Google say? 
 PDF only 
| .edu only 

---

**Interactive relationship** (e.g. stimulation, inhibition, etc)

**Neutral relationship**- Lectins interact differentially with purified human eosinophils, cultured cord blood derived mast cells and the myeloid leukaemic cell line AML14.3D10 induction of interleukin 4  [ **IL4R** ]   [ **IL4** ]  secretion is conserved among granulocytes, but is not proportional to agglutination or lectin glycoprotein interaction.  Ref: 12859449 Clin Exp Allergy, 2003

**Non-interactive relationship** (e.g. studied together, co-existance, homology, etc.)

- Cytokine profiles patterns in the expression of interleukin 4  [ **IL4R** ]   [ **IL4** ]  IL 4  [ **IL4** ] , interleukin 10, interleukin 12, interferon gamma IFN gamma and tumour necrosis factor alpha TNF alpha were therefore studied during the natural acquisition of immunity to Plasmodium falciparum and P. vivax among individuals from Buenaventura, a meso endemic region on the Pacific Coast of Colombia.  Ref: 12831518 Ann Trop Med Parasitol, 2003
- T cells mediate an inhibitory effect of interleukin 4  [ **IL4R** ]   [ **IL4** ]  on osteoclastogenesis.  Ref: 12817750 J Bone Miner Res, 2003
- Co immobilization of antibodies to CD3 and CD4 enhanced cell recoveries and induced interleukin 4  [ **IL4R** ]   [ **IL4** ]  as well as interferon gamma synthesis.  Ref: 12848849 Immunol Cell Biol< MedlineTA>Immunol Cell Biol, 2003
- Expression of transferrin receptors, type 1 insulin like growth factor receptors IGF1R, and interleukin 4  [ **IL4R** ]   [ **IL4** ]  receptors  **IL4R**  was measured by flow cytometry analysis on unirradiated cells and on cells 3 to 120 h after irradiation.  Ref: 12859234 Radiat Res, 2003
- With splenocytes from vaccinated mice, specific T cell responses, as well as gamma interferon and interleukin 4  [ **IL4R** ]   [ **IL4** ]  production, were observed after stimulation with P. chabaudi adami infected erythrocytes, demonstrating the specificity of genomic library vaccination for two of the three libraries constructed.  Ref: 12874330 Infect ImmunInfect Immun, 2003
- Naive T cells from transgenic mice expressing a dominant interfering form of Runx1 exhibited enhanced interleukin 4  [ **IL4R** ]   [ **IL4** ]  production and efficient Th2 differentiation.  Ref: 12835475 J Exp Med, 2003
- Prevention of brain metastasis formation by local expression of interleukin 4  [ **IL4R** ]   [ **IL4** ]  or hemagglutinin antigen.  Ref: 12838474 Zentralbl Neurochir, 2003
- There are many candidate genes, particular the cluster of interleukin 4  [ **IL4R** ]   [ **IL4** ]  genes 5q31 33, the major histocompatibility complex genes HLA D and the TNFalpha gene 6p21 23, the gene of the receptor B chain with strong affinity for IgE 11q13, the interferon gamma gene 12q15 24, the genes of the alpha gamma chains of the T receptor 14q11, the  **IL4**  receptor gene 16p12, and the metalloprotease gene ADAM33 20p13.  Ref: 12843991 Rev Pneumol Clin, 2003
- METHODS Interferon gamma IFN gamma and interleukin 4  [ **IL4R** ]   [ **IL4** ]  IL 4  [ **IL4** ]  secreted by CD4 T cells were detected by flow cytometry in 40 patients with CA and 20 healthy volunteers, and significance of Th1 Th2 cell ratio in the etiology of the CA was analyzed.  Ref: 12865237 Di Yi Jun Yi Da Xue Xue Bao, 2003
- Real time polymerase chain reaction studies revealed significantly higher interleukin 4  [ **IL4R** ]   [ **IL4** ]  p< ; 0.05 and transforming growth factor beta p< ; 0.05 gene expression in the gastric mucosa in infected mice than in uninfected mice at both two and six months after AIG induction.  Ref: 12865266 Gut, 2003
- The roles of interleukin 4  [ **IL4R** ]   [ **IL4** ]  IL 4  [ **IL4** ]  and IL 13 in the regulation of immunity to Leishmania donovani infection are still poorly understood.  Ref: 12874364 Infect ImmunInfect Immun, 2003
- Endogenous interleukin 4  [ **IL4R** ]   [ **IL4** ]  and interleukin 10 regulate experimental lung ischemia reperfusion injury.  Ref: 12842552 Ann Thorac Surg, 2003
- Interleukin 4  [ **IL4R** ]   [ **IL4** ]  IL 4  [ **IL4** ]  is crucial for the differentiation of naive T helper T H cells into the T H 2 effector cells that promote humoral antibody immunity and provide protection against intestinal helminths.  Ref: 12876556 Nat Rev Immunol, 2003
- Granulocyte monocyte colony stimulating factor, interleukin 4  [ **IL4R** ]   [ **IL4** ] , and CD40 ligand in combination are capable of yielding dendritic cells from at least some cases of acute lymphocytic leukemia.  Ref: 12850495 Blood Cells Mol Dis< MedlineTA>Blood Cells Mol Dis, 2003
- Changes in Expression of Transferrin, Insulin like Growth Factor 1, and Interleukin 4  [ **IL4R** ]   [ **IL4** ]  Receptors after Irradiation of Cells of Primary Malignant Brain Tumor Cell Lines.  Ref: 12859234 Radiat Res, 2003
- Both Interleukin 4  [ **IL4R** ]   [ **IL4** ]  IL 4  [ **IL4** ]  and IL 4  [ **IL4** ]  Receptor alpha Signaling Contribute to the Development of Hepatic Granulomas with Optimal Antileishmanial Activity.  Ref: 12874364 Infect ImmunInfect Immun, 2003
- undetectable interleukin 4  [ **IL4R** ]   [ **IL4** ]  and interleukin 10.  Ref: 12891535 Gastroenterology, 2003
- In this article, we review recent data regarding the role of functional polymorphisms in the genes encoding the prototypic Th1 cytokine interferon gamma and Th2 cytokine interleukin 4  [ **IL4R** ]   [ **IL4** ]  in multifactorial disorders.  Ref: 12823954 Trends Pharmacol Sci, 2003
- Overexpression of Interleukin 4  [ **IL4R** ]   [ **IL4** ]  in Lungs of Mice Impairs Elimination of Histoplasma capsulatum.  Ref: 12819061 Infect Immun, 2003
- The data suggest that interleukin 4  [ **IL4R** ]   [ **IL4** ]  independent priming of interleukin 4  [ **IL4R** ]   [ **IL4** ]  producing cells in this system requires sustained stimulation via both the T cell receptor and CD4 with close physical association between the ligands for these two receptors.  Ref: 12848849 Immunol Cell Biol< MedlineTA>Immunol Cell Biol, 2003
- In cultured FRC, TG is induced 5 to 20 fold and becomes colocalized with matrix proteins by treatment with interleukin 4  [ **IL4R** ]   [ **IL4** ] , but not by other cytokines that are commonly increased in lymph upon immune activation.  Ref: 12819021 Am J Pathol, 2003
- Most strikingly, there was a sharp increase in gamma interferon IFN gamma production by schistosome egg antigen stimulated granuloma cells, bulk mesenteric lymph node MLN cells, and purified MLN CD4 T cells, which contrasted with a more discreet change in the Th2 type cytokines interleukin 4  [ **IL4R** ]   [ **IL4** ]  IL 4  [ **IL4** ]  and IL 10.  Ref: 12819093 Infect Immun, 2003
- Interferon gamma IFN gamma, interleukin 4  [ **IL4R** ]   [ **IL4** ]  IL 4  [ **IL4** ] , adhesion molecules, and activation markers were detected by immunohistochemistry, and expression of IFN gamma and IL 4  [ **IL4** ]  messenger RNA was revealed by in situ hybridization.RESULTS uFA patients had a higher density of IFN gamma positive cells in the lamina propria than did tFA patients and controls P = 0.053 and P = 0.018.  Ref: 12827002 J Pediatr Gastroenterol Nutr, 2003
- Interleukin 4  [ **IL4R** ]   [ **IL4** ]  stimulation of wild type cells resulted in a 2 3 fold increase in Stat 6 phosphorylation.  Ref: 12848851 Immunol Cell Biol, 2003
- Replicating DC progenitor of C57BL 6 mice was transduced by retroviral vector and continually cultured in the presence of recombinant mouse granulocyte macrophage colony stimulating factor rmGM CSF and interleukin 4  [ **IL4R** ]   [ **IL4** ]  IL 4  [ **IL4** ]  for 6 days.  Ref: 12854153 World J Gastroenterol, 2003
- Previous work has shown that CD4 engagement can promote the development of interleukin 4  [ **IL4R** ]   [ **IL4** ]  producing cells from naive CD4 T cells activated with anti CD3 antibody and interleukin 2 in the absence of other exogenous signals, including interleukin 4  [ **IL4R** ]   [ **IL4** ]  itself.  Ref: 12848849 Immunol Cell Biol< MedlineTA>Immunol Cell Biol, 2003
- Using an in vitro cell culture system, we investigated how the key regulatory cytokines interleukin 4  [ **IL4R** ]   [ **IL4** ]  IL 4  [ **IL4** ] , IL 10, IL 13, and TGF beta might modulate the effect of IFN gamma in inducing resistance to infection in enterocyte cell lines.  Ref: 12874337 Infect Immun, 2003
- These studies were undertaken to investigate the role of two regulatory cytokines, interleukin 4  [ **IL4R** ]   [ **IL4** ]  IL 4  [ **IL4** ]  and IL 10, in an in vivo model of LIRI.Left lungs of Long Evans rats underwent normothermic ischemia for 90 minutes and reperfusion for up to 4 hours.  Ref: 12842552 Ann Thorac Surg, 2003
- These signals initiate reciprocal activation and silencing of the interferon gamma IFN gamma and interleukin 4  [ **IL4R** ]   [ **IL4** ]  IL 4  [ **IL4** ]  cytokine gene loci, changes that are heritably maintained in the resulting T helper type 1 T H 1 or T H 2 cells and their progeny.  Ref: 12830136 Nat Immunol, 2003
- No interleukin 4  [ **IL4R** ]   [ **IL4** ]  mRNA was detected in either subpopulation.  Ref: 12827664 Cytometry, 2003
- Interleukin 4  [ **IL4R** ]   [ **IL4** ]  induces Coxiella burnetii replication in human monocytes but not in macrophages.  Ref: 12860673 Ann N Y Acad Sci, 2003
- Effects of intrinsic nitric oxide on the expression of interleukin 4  [ **IL4R** ]   [ **IL4** ]  and IFN gamma mRNA in the bronchial and lung tissues of sensitized rats.  Ref: 12845750 J Tongji Med Univ, 2000
- In vitro culture of bone marrow BM cells with granulocyte macrophage colony stimulating factor and interleukin 4  [ **IL4R** ]   [ **IL4** ]  induced a dramatic increase of telomerase activity accompanied with their differentiation into DCs.  Ref: 12885944 J Leukoc Biol, 2003
- A molecule solves psoriasis ? Systemic therapies for psoriasis inducing interleukin 4  [ **IL4R** ]   [ **IL4** ]  and Th2 responses.  Ref: 12879154 J Mol MedJ Mol Med, 2003
- In view of the importance of interleukin 4  [ **IL4R** ]   [ **IL4** ]  IL 4  [ **IL4** ]  gene as a genetic factor for adult periodontitis, we investigated the relationship between two polymorphisms 590 C > T polymorphism and 70 bp repeat polymorphism of the human IL 4  [ **IL4** ]  gene and adult periodontitis in the Korean population.  Ref: 12877559 Arch Pharm Res, 2003
- Interleukin 4  [ **IL4R** ]   [ **IL4** ]  levels significantly decreased P <.  Ref: 12877453 Ann Allergy Asthma Immunol, 2003
- In vitro stimulation of lymphocytes from both Th1 biased C57Bl 6 and T cell receptor transgenic mice and Th2 biased BALB c mice in the presence of OdDHL demonstrated that OdDHL inhibits in vitro cytokine production in response to both mitogen and antigen, with gamma interferon IFN gamma tending to be more inhibited than interleukin 4  [ **IL4R** ]   [ **IL4** ]  IL 4  [ **IL4** ] .  Ref: 12874321 Infect Immun, 2003
- Changes in Expression of Transferrin, Insulin like Growth Factor 1, and Interleukin 4  [ **IL4R** ]   [ **IL4** ]  Receptors after Irradiation of Cells of Primary Malignant Brain Tumor Cell Lines.  Ref: 12859234 Radiat Res, 2003
- Role of Interleukin 4  [ **IL4R** ]   [ **IL4** ]  in Resistance to Cryptococcus neoformans Infection.  Ref: 12855407 Am J Respir Cell Mol Biol< MedlineTA>Am J Respir Cell Mol Biol, 2003
- Wheat gliadin promotes the interleukin 4  [ **IL4R** ]   [ **IL4** ]  induced IgE production by normal human peripheral mononuclear cells through a redox dependent mechanism.  Ref: 12824000 Cytokine, 2003
- In the present study, we have employed DNA immunization protocols using Sm p80 with plasmids encoding granulocyte macrophage colony stimulating factor GM CSF and interleukin 4  [ **IL4R** ]   [ **IL4** ]  IL 4  [ **IL4** ] .  Ref: 12819068 Infect Immun< MedlineTA>Infect Immun, 2003
- Two polymorphisms of interleukin 4  [ **IL4R** ]   [ **IL4** ]  gene in Korean adult periodontitis.  Ref: 12877559 Arch Pharm Res, 2003
- APN CD13 was significantly upregulated by treatment with interleukin 4  [ **IL4R** ]   [ **IL4** ]  IL 4  [ **IL4** ] , interferon gamma IFNgamma, and the glucocorticoids dexamethasone and hydrocortisone.  Ref: 12861419 Arch Dermatol ResArch Dermatol Res, 2003
- When examined in the presence of both CD154 and interleukin 4  [ **IL4R** ]   [ **IL4** ] , Tax had a clear supportive role on B cell growth, with an impact on B cell proliferation, cell cycle phase distribution, and survival.  Ref: 12881710 Oncogene, 2003
- When CD44low CD4 T cells were activated with immobilized anti CD3 antibody and interleukin 2, they proliferated and produced interferon gamma but not interleukin 4  [ **IL4R** ]   [ **IL4** ] .  Ref: 12848849 Immunol Cell Biol< MedlineTA>Immunol Cell Biol, 2003
- Interleukin 4  [ **IL4R** ]   [ **IL4** ]  IL 4  [ **IL4** ]  is a major cytokine regulating IgE production and IgE response.  Ref: 12872985 Mol Cells, 2003
- Aim To measure serum concentrations of the soluble interleukin 2 receptor sIL2R, soluble interleukin 4  [ **IL4R** ]   [ **IL4** ]  receptor sIL4R, soluble interleukin 6 receptor sIL6R, and soluble tumor necrosis factor alpha receptor I and soluble tumor necrosis factor alpha receptor II, during the perinatal and early neonatal period, in order to evaluate their role in activation of immune response in labor and the first days postpartum.  Ref: 12857603 Mediators Inflamm, 2003
- Feasibility to generate monocyte derived dendritic cell from coculture with melanoma tumor cells in the presence of granulocyte macrophage colony stimulating factor GM CSF and interleukin 4  [ **IL4R** ]   [ **IL4** ] .  Ref: 12852497 Am J Reprod Immunol, 2003
- In the ceramic on ceramic group, leukocytes and lymphocyte subsets were not significantly changed, but a significant increase in type 2 cytokines restored the ratio of interferon gamma to interleukin 4  [ **IL4R** ]   [ **IL4** ]  to normal values.  Ref: 12892206 J Bone Joint Surg Br, 2003
- In order to measure the plasma levels of interleukin 4  [ **IL4R** ]   [ **IL4** ]  IL 4  [ **IL4** ] , cyclic adenosine monophosphate cAMP and cyclic guanosine monophosphate cGMP in patients with chronic obstructive pulmonary disease COPD and observe the effects of oral theophylline on them, we divided 28 COPD patients into COPD experimental group and COPD control group.  Ref: 12840867 J Tongji Med Univ, 1999
- Interleukin 4  [ **IL4R** ]   [ **IL4** ]  and Transforming Growth Factor beta Have Opposing Regulatory Effects on Gamma Interferon Mediated Inhibition of Cryptosporidium parvum Reproduction.  Ref: 12874337 Infect Immun, 2003
- Both interleukin 4  [ **IL4R** ]   [ **IL4** ]  and CD40 ligand are important for optimal dendritic cell differentiation and maturation.  Ref: 12850495 Blood Cells Mol Dis< MedlineTA>Blood Cells Mol Dis, 2003
- Neutralizing antibodies against human RANTES, MIP 1alpha, MIP 1beta, alpha interferon IFN alpha, IFN beta, IFN gamma, interleukin 4  [ **IL4R** ]   [ **IL4** ]  IL 4  [ **IL4** ] , IL 10, IL 13, IL 16, MCP 1, MCP 3, tumor necrosis factor alpha TNF alpha, or TNF beta failed to reverse the HIV 1 suppressive activity.  Ref: 12885891 J VirolJ Virol, 2003
- Dendritic cells were derived from CD34 cells with culture in GM CSF, tumor necrosis factor alpha, and serum free media or from monocytes with culture in GM CSF, interleukin 4  [ **IL4R** ]   [ **IL4** ] , and autologous serum.  Ref: 12857369 J Hematother Stem Cell Res, 2003
- Gene therapy with GM CSF, interleukin 4  [ **IL4R** ]   [ **IL4** ]  and herpes simplex virus thymidine kinase shows strong antitumor effect on lung cancer.  Ref: 12828142 Anticancer Res< MedlineTA>Anticancer Res, 2003
- The proliferation was more pronounced and lasted longer as compared with other activators of B cells, such as anti IgM in the presence of interleukin 4  [ **IL4R** ]   [ **IL4** ]  or lipopolysaccharide.  Ref: 12832451 J Leukoc Biol, 2003
- To assess their utility, activation of the B cell receptor using anti micro, the Toll like receptor 4 using lipopolysaccharide and the interleukin 4  [ **IL4R** ]   [ **IL4** ]  receptor were assessed in these cell lines.  Ref: 12848851 Immunol Cell Biol, 2003
- Since interleukin 4  [ **IL4R** ]   [ **IL4** ]  IL 4  [ **IL4** ]  can inhibit both Th1 cytokine production and activity, we examined the effects of overproduction of IL 4  [ **IL4** ]  in the lung on the course of pulmonary histoplasmosis.  Ref: 12819061 Infect Immun, 2003
- Effects of theophylline on plasma levels of interleukin 4  [ **IL4R** ]   [ **IL4** ] , cyclic nucleotides and pulmonary functions in patients with chronic obstructive pulmonary disease.  Ref: 12840867 J Tongji Med Univ, 1999
- Removal of the anti CD4 antibodies within the first three days of stimulation also prevented the development of detectable interleukin 4  [ **IL4R** ]   [ **IL4** ]  producing cells.  Ref: 12848849 Immunol Cell Biol< MedlineTA>Immunol Cell Biol, 1999
- Induction of Protective Immunity against Schistosoma mansoni via DNA Priming and Boosting with the Large Subunit of Calpain Sm p80 Adjuvant Effects of Granulocyte Macrophage Colony Stimulating Factor and Interleukin 4  [ **IL4R** ]   [ **IL4** ] .  Ref: 12819068 Infect Immun< MedlineTA>Infect Immun, 1999
- Interleukin 4  [ **IL4R** ]   [ **IL4** ]  and IL 13 activated phosphorylation of mitogen activated protein kinase in LS174T cells.  Ref: 12848848 Immunol Cell Biol, 2003
- To investigate the effects of intrinsic nitric oxide NO on the expression of interleukin 4  [ **IL4R** ]   [ **IL4** ]  IL 4  [ **IL4** ]  mRNA and interferon gamma IFN gamma mRNA in the airway inflammation of asthma, the rat models of asthmatic inflammation were established by sensitizing and then challenging the animals with ovalbumin.  Ref: 12845750 J Tongji Med Univ, 2000
- By contrast, inhalation of a soluble analogue E861, K875 of peptide 861 874 deviated the autoimmune response towards a Th2 profile, with marked increases in the ratio of interleukin 4  [ **IL4R** ]   [ **IL4** ]  to interferon gamma produced by splenic T cells responding in vitro to either peptide 861 874 or Band 3.  Ref: 12829598 Blood< MedlineTA>Blood, 2000
- The tumor necrosis factor alpha TNF alpha, interleukin 4  [ **IL4R** ]   [ **IL4** ]  IL 4  [ **IL4** ]  levels and liver function before and after PE therapy were examined.  Ref: 12857473 Zhongguo Wei Zhong Bing Ji Jiu Yi Xue, 2003
- Before THR, the patients showed a decrease in leukocytes and myeloid cells in comparison with healthy donors, and a prevalence of type 1 T lymphocytes, which was confirmed by the increase in ratio of interferon gamma to interleukin 4  [ **IL4R** ]   [ **IL4** ] .  Ref: 12892206 J Bone Joint Surg Br, 2003
